# Esketamine combined with sufentanil *versus* sufentanil in patient-controlled intravenous analgesia: a meta-analysis

**DOI:** 10.3389/fphar.2024.1247646

**Published:** 2024-02-07

**Authors:** Manman Yao, Baoxia Fang, Jinguo Yang, Peng Chen, Fuchao Chen

**Affiliations:** ^1^ Sinopharm Dongfeng General Hospital, Hubei University of Medicine, Shiyan, Hubei, China; ^2^ School of Pharmaceutical Sciences, Hubei University of Medicine, Shiyan, Hubei, China; ^3^ Renmin Hospital of Wuhan University, Wuhan, Hubei, China

**Keywords:** patient-controlled intravenous analgesia, sufentanil, esketamine, postoperative pain, meta-analysis

## Abstract

**Objective:** Patient-controlled intravenous analgesia (PCIA) can alleviate pain to some extent, and several randomized controlled trials (RCTs) have examined the efficacy of esketamine-assisted sufentanil in postoperative PCIA. In this research, we conducted a meta-analysis of relevant RCTs to compare the effect and safety of esketamine-sufentanil *versus* sufentanil alone for postoperative PCIA.

**Methods:** We systematically searched the Cochrane Library, PubMed, Embase, Web of Science, CNKI, and other libraries up to December 2023 to screen out RCTs examining the use of esketamine combined with sufentanil for PCIA. We analysed analgesia scores, sedation scores, adverse drug reactions and *postpartum* depression scores as outcome indicators.

**Results:** This meta-analysis included 32 RCTs. The results of the meta-analysis were as follows. 1) Visual Analog Scale: The VAS scores at 6, 12, 24, and 48 h were lower in the esketamine-sufentanil group than in the sufentanil alone group, and significant differences were found at all time points (*p* < 0.05). 2) Ramsay Sedation Scale: The sedation score of the esketamine-sufentanil group at 48 h after surgery was higher than that of the sufentanil group alone [mean difference (MD) = −0.09 points, confidence interval (*CI*): (−0.26, −0.07), *p* = 0.27], but this difference was not significant (*p* > 0.05). 3) Safety: Compared with sufentanil alone, the incidence rates of postoperative nausea-vomiting, dizziness-headache, skin pruritus and respiratory depression were significantly lower in the esketamine-sufentanil group. 4) *Postartum* depression: The reduction in *postpartum* depression scores were significantly greater in the esketamine-sufentanil group than in the sufentanil alone group at 3 days [MD = −1.35 points, *CI*: (−1.89, −0.81), *p* < 0.00001] and 7 days [MD = −1.29 points, *CI*: (−2.42, −0.16), *p* = 0.03].

**Conclusion:** The meta-analysis showed that the use of esketamine combined with sufentanil for postoperative PCIA could improve postoperative analgesia, alleviate *postpartum* depression and reduce the rate of postoperative adverse reactions, but there was no significant difference in sedation.

## 1 Introduction

Postoperative pain is a reaction to tissue damage in patients undergoing surgery. Effective postoperative analgesia can not only reduce patients’ pain but also decrease postoperative complications and enhance patients’ recovery, which are intrinsic requirements for rapid postoperative recovery (Barratt et al., 2021). Patient-controlled intravenous analgesia (PCIA) is a widely recognized method of postoperative analgesia that combines two or more analgesic drugs to produce a synergistic effect by acting on different targets. It not only reduces the dosage of analgesic drugs but also enhances their analgesic effect. Among the various drugs used for postoperative pain, opioids are commonly used as analgesics, as they can effectively control pain. (Shanthanna et al., 2021). However, the use of opioids may cause adverse reactions, including postoperative nausea and vomiting (PONV), respiratory depression, intestinal obstruction, delirium, and pain sensitivity, thereby significantly increasing patient pain and prolonging hospital stays (Bicket et al., 2017). Therefore, some clinical guidelines recommend combinations of analgesic drugs for PCIA to provide effective pain relief while reducing opioid-induced adverse reactions and related risks (Li et al., 2020).

Esketamine, which is the S (+) isomer of ketamine, acts on the N-methyl-D-aspartic acid (NMDA) receptor and plays an anaesthetic role (Zanos et al., 2018a). Its drugging effect is nearly twice as great as that of (R,S)-ketamine and three times greater than that of (R)-ketamine (Wang et al., 2019). Research has shown that a low dose of esketamine can reduce the incidence of anaesthesia-related side effects and has good analgesic effects, fewer adverse reactions, a short recovery time, and antidepressant effects (Hamp et al., 2018; Shoib et al., 2022). Therefore, esketamine is widely used in clinical practice. Sufentanil, which is a derivative of fentanyl, is a powerful µ-opioid receptor agonist that has the advantages of rapid onset, stable haemodynamics, and few side effects. It is currently an ideal postoperative analgesic drug (Qu and Wu, 2022). In recent years, sufentanil has become popular in clinical practice for PCIA. Thus, we performed a meta-analysis of randomized controlled trials (RCTs) on sufentanil and esketamine to quantify treatment with esketamine as the adjuvant for PCIA after sufentanil surgery.

## 2 Methods

The data in this study were collected and analysed in accordance with the guidelines published by the Cochrane Society and the Preferred Reporting Items for Systematic Reviews and Meta-Analyses (PRISMA) guidelines (Liberati et al., 2009). Ethics approval and informed consent were not required because this meta-analysis was a summary of prior research.

### 2.1 Search strategy

In this review, two researchers independently searched the PubMed, Web of Science, Embase, Cochrane Library, China National Knowledge Infrastructure (CNKI), Wanfang and VIP databases from inception to December 2023, to retrieve potentially relevant literature. The search was conducted by combining free words with subject words, and the search terms included “esketamine,” “sufentanil,” “s-ketamine,” “esketamine hydrochloride,” “sufentanil citrate,” “intravenous” and “analgesia” using the Boolean operators “AND or OR”. RCTs examining the use if esketamine and sufentanil or the use of sufentanil alone for postoperative PCIA were considered for potential inclusion. To prevent the omission of literature, additional relevant references were manually searched. Then, two authors, Manman Yao and Baoxia Fang, independently searched the literature and extracted the data. Disagreements regarding study inclusion were resolved by discussion or by consulting a third party.

### 2.2 Study inclusion and exclusion criteria

The inclusion criteria were as follows: 1) Study design: RCTs; 2) Subjects: adult surgical patients who used intravenous PCIA after surgery; 3) Intervention: esketamine combined with sufentanil for PCIA; 4) Comparison: sufentanil alone for PCIA; 5) Outcome measures: at least one of the following outcomes: VAS score, RSS score, postoperative adverse reactions (nausea, vomiting, dizziness, headache, pruritus, and respiratory depression) and postpartum depression score.

The exclusion criteria were as follows: 1) non-RCTs; 2) patients with single medication or combined medication but no PCIA after surgery; 3) studies of postoperative dural self-controlled analgesia combined with medication; 4) studies with no control group or without sufentanil; 5) studies without the original text, studies with incomplete data, or duplicate studies.

### 2.3 Data extraction

The literature was screened independently by two authors, and the data were extracted and cross-checked. Disagreements were resolved by discussion or by consulting a third party. First, the titles were screened, followed by reading the abstracts and full texts to exclude clearly unrelated references. If necessary, the reviewers contacted the authors of the original study by email or phone to obtain additional information that not extracted from the publication. The following data were extracted: 1) essential information about the study, including the title of the study, primary author, journal of delivery, etc. 2) baseline characteristics of participants and interventions, including sample size, type of surgery, drug use, etc. 3) critical information for evaluating the risk of bias; and 4) outcome indicators and outcome measures.

### 2.4 Quality assessment

After collecting relevant literature that met the eligibility criteria, the two authors independently analysed the included studies. The risk of bias of each included study was evaluated across seven domains using the Cochrane Risk of Bias Tool (Higgins et al., 2011). For each study, the domains were categorized as high risk of bias, unclear risk of bias, or low risk of bias. Disagreements between the two reviewers were resolved by discussion or by consulting a third party.

### 2.5 Statistical analysis

Quantitative analysis of the included data was performed using Review Manager 5.4. The mean difference (MD) of measurement data was used as the statistic of effect analysis, and the risk difference (RD) or odds ratio (OR) of dichotomous variables was the effect size measure (combined with the corresponding 95% *CIs*). Heterogeneity between the included studies was evaluated using the *χ* ^2^ test and *I* ^2^ statistic. *p* > 0.1 and *I* ^2^ < 50% indicated low heterogeneity, and in such cases, a fixed effects model was used. *p* < 0.1 and *I* ^2^ > 50% indicated high heterogeneity, and in such cases, a random effects model was used.

A sufficient number of studies were included in this study (n > 10), so a publication bias analysis was conducted. If necessary, sensitivity analyses were performed to determine the source of heterogeneity by excluding studies one at a time. *p* < 0.05 indicated statistically significant differences. In the included studies, esketamine was widely used in caesarean sections. Through comprehensive analysis and expert advice, the study analysed the effects of esketamine on depression after caesarean delivery.

## 3 Results

### 3.1 Study selection and characteristics of studies

**FIGURE 1:**
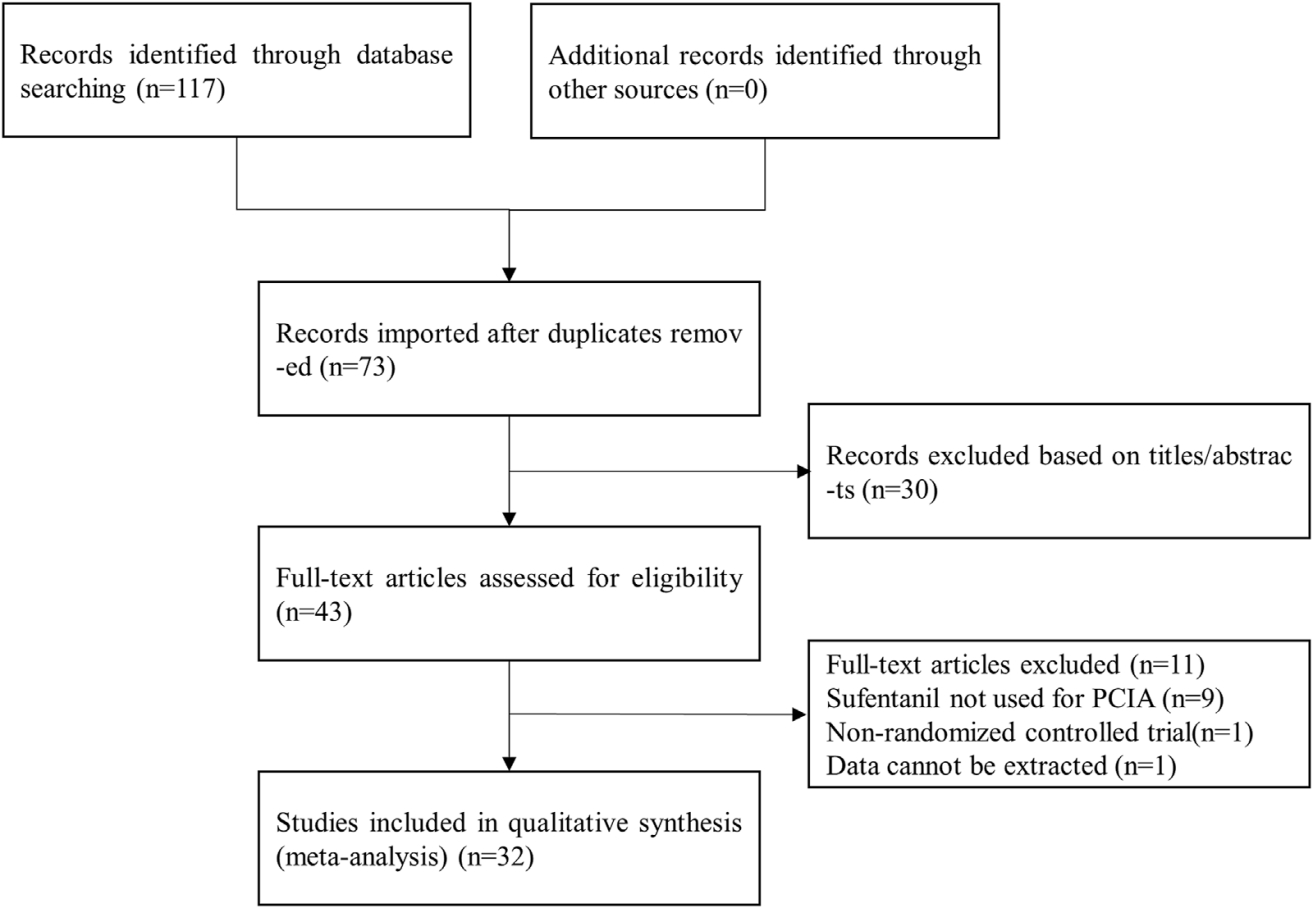
Study flow chart.

In this paper, 117 relevant studies were initially retrieved after using the keywords to search the databases. Then, 44 duplicate publications were excluded, and 73 studies remained for preliminary screening. After further reading the titles and abstracts of the remaining articles, 30 studies that did not meet the standards were excluded, and the remaining 43 studies were subjected to full-text screening. A total of 9 papers that examined PCIA without sufentanil were excluded, 1 nonrandomized controlled trial was excluded, and 1 study with incomplete data was excluded. Ultimately, 32 valid studies were included in this meta-analysis, including 3,709 patients (Guo et al., 2021; Li, 2021; Lyu et al., 2021; Yan, 2021; Wang JF. et al., 2022; Wang N. et al., 2022; Cai et al., 2022; Chi et al., 2022; Wang Y. et al., 2022; Wang W. et al., 2022; Han et al., 2022; He et al., 2022; Jiang et al., 2022; Li et al., 2022; Liang, 2022; Luo et al., 2022; Peng et al., 2022; Qiu et al., 2022; Qiu and Wang, 2022; Wang, 2022; Xu and Li, 2022; Zhang et al., 2022; Zheng et al., 2022; Zhou et al., 2022; Yang B. et al., 2023; Zhang et al., 2023a; Yang SQ. et al., 2023; Gui et al., 2023; Han et al., 2023; Su et al., 2023; Wang et al., 2023; Xie et al., 2023) (Figure 1).

### 3.2 Study characteristics

This meta-analysis included 32 RCTs, and the included studies were published between 2016 and 2023. The characteristics of the included studies are shown in Table 1.

**TABLE 1:** Characteristics of included trials.

Trials (year)	Country	Age	Group	Drug concentration (PCIA)		Sugery	Time (h)	Outcomes
				Sufentanil	ESK			
HL Liang 2022	China	21–37	Control (*n* = 38)	3 µg/kg	2 mg/kg	Caesarean section	48	1,4
			ESK + sufentanil (*n* = 38)	2 µg/kg				
HY Jiang 2022	China	24–31	Control (*n* = 30)	2 µg/kg	2 mg/kg	Caesarean section	48	1,2,4
			ESK + sufentanil (*n* = 30)	2 µg/kg				
XW Chi 2022	China	24–45	Control (n = 56)	3 µg/kg	2 mg/kg	Caesarean section	48	1,2,3
			ESK + sufentanil (n = 56)	2 µg/kg				
L Cai 2022	China	18–65	Control (*n* = 43)	2.5 µg/kg	2 mg/kg	Hip replacement	72	1,2,4
			ESK + sufentanil (*n* = 43)	1.25 µg/kg				
SG Wang 2022	China	21⁓37	Control (*n* = 200)	3 µg/kg	2 mg/kg	Caesarean section	48	1,2,3,4
			ESK + sufentanil (*n* = 200)	2 µg/kg				
JF Wang 2022	China	18–65	Control (*n* = 60)	2 µg/kg	1.5 mg/kg	thoracic surgery	48	4
			ESK + sufentanil (*n* = 60)	1 µg/kg				
XY Zhang 2022	China	25–40	Control (*n* = 60)	1.5 µg/kg	1.5 mg/kg	Mixed hemorrhoids	72	1
			ESK + sufentanil (*n* = 61)	1.5 µg/kg				
SG Ly 2022	China	64–72	Control (*n* = 50)	1.5 µg/kg	1.44 mg/kg	abdominal operation	48	1,2,4
			ESK + sufentanil (*n* = 50)	1.05 µg/kg				
Y Su 2023	China	39–80	Control (n = 34)	1.5 µg/kg	1.2 mg/kg	thoracic surgery	48	1,2,4
			ESK + sufentanil (n = 33)	1 µg/kg				
F Xie 2023	China	41–69	Control (*n* = 52)	2 µg/kg	1 mg/kg	abdominal operation	48	1,4
			ESK + sufentanil (*n* = 52)	1.5 µg/kg				
N Wang 2022	China	66–80	Control (*n* = 40)	100 µg	1 mg/kg	Esophageal cancer radical surgery	48	1,2
			ESK + sufentanil (*n* = 40)	50 µg				
F Qiu 2022	China	50–75	Control (n = 30)	2 µg/kg	1 mg/kg	spinal fusion	48	1,2,4
			ESK + sufentanil (n = 30)	1.5 µg/kg				
WF Gui 2023	China	18–70	Control (*n* = 32)	2 µg/kg	0.5 mg/kg	thoracic surgery	48	4
			ESK + sufentanil (*n* = 34)	2 µg/kg				
J Li 2022	China	34–67	Control (*n* = 45)	2 µg/kg	0.5 mg/kg	radical mastectomy w	48	1
			ESK + sufentanil (*n* = 45)	2 µg/kg				
JG Zheng 2022	China	18–45	Control (*n* = 70)	1 µg/kg	0.5 mg/kg	Caesarean section	48	1
			ESK + sufentanil (*n* = 70)	1 µg/kg				
YQ Han 2022	China	18–45	Control (n = 153)	2 µg/kg	0.5 mg/kg	Caesarean section	48	4
			ESK + sufentanil (n = 122)	2 µg/kg				
Y Wang 2022	China	25–35	Control (*n* = 132)	50 µg	0.2–0.5 mg/kg	Caesarean section	48	4
			ESK + sufentanil (*n* = 108)	50 µg				
PL Li 2021	China	20–40	Control (*n* = 153)	2 µg/kg	0.5 mg/kg	Caesarean section	48	1,2,3
			ESK + sufentanil (*n* = 122)	2 µg/kg				
B Yang 2023	China	44–57	Control (*n* = 40)	2 µg/kg	0.36 mg/kg	abdominal operation	48	1,4
			ESK + sufentanil (*n* = 40)	2 µg/kg				
JM Yan 2016	China	20–24	Control (n = 50)	1 µg/kg	0.36 mg/kg	Caesarean section	48	1,3,4
			ESK + sufentanil (n = 50)	0.5 µg/kg				
Y Peng 2022	China	18–64	Control (*n* = 18)	2 µg/kg	0.25 mg/kg	Spinal orthopedics	72	2,4
			ESK + sufentanil (*n* = 18)	1.5 µg/kg				
W Wang 2022	China	22–35	Control (n = 39)	1.5 µg/kg	0.2 mg/kg	Caesarean section	48	4
			ESK + sufentanil (n = 40)	1.5 µg/kg				
P Zhou 2022	China	65–82	Control (*n* = 30)	200 µg	100 mg	Hip replacement	48	1,2
			ESK + sufentanil (*n* = 30)	100 µg				
QM Qiu 2022	China	35–62	Control (*n* = 30)	3 µg/kg	50 mg	general anesthesia	48	1,2,4
			ESK + sufentanil (*n* = 30)	3 µg/kg				
J Guo 2021	China	20–35	Control (*n* = 56)	100 µg	50 mg	Caesarean section	24	1,2,3
			ESK + sufentanil (*n* = 56)	100 µg				
R He 2022	China	>18	Control (n = 41)	100 µg	45 mg	Caesarean section	48	3
			ESK + sufentanil (n = 41)	50 µg				
YF Luo 2022	China	50–69	Control (*n* = 30)	2 µg/kg	0.03 mg/kg/h	thoracic surgery	48	1,4
			ESK + sufentanil (*n* = 30)	2 µg/kg				
N Xu 2022	China	18–70	Control (*n* = 30)	0.03 µg/kg/h	25 µg/kg/h	Open reduction of fracture	48	1,4
			ESK + sufentanil (*n* = 30)	0.02 µg/kg/h				
M Wang 2023	China	18–80	Control (*n* = 43)	100 µg	1.25 mg/kg	breast cancer surgery	48	3
			ESK + sufentanil (*n* = 43)	100 µg				
TP Zhang 2023	China	18–65	Control (*n* = 42)	2 µg/kg	50 mg	abdominal surgery	48	1,3
			ESK + sufentanil (*n* = 44)	2 µg/kg				
T Han 2023	China	27–37	Control (*n* = 70)	150 µg	0.5 mg/kg	Caesarean section	48	1,3
			ESK + sufentanil (*n* = 70)	150 µg				
SQ Yang 2023	China	18–80	Control (*n* = 97)	2.2 µg/kg	2 mg/kg	Caesarean section	48	1,3
			ESK + sufentanil (*n* = 99)	2.2 µg/kg				

1 Postoperative VAS, pain score.

2 Postoperative RSS, sedation score.

3 The incidence of postoperative adverse reactions.

4 Postoperative EPDS, score; ESK = esketamine.

### 3.3 Risk of bias assessment

**FIGURE 2:**
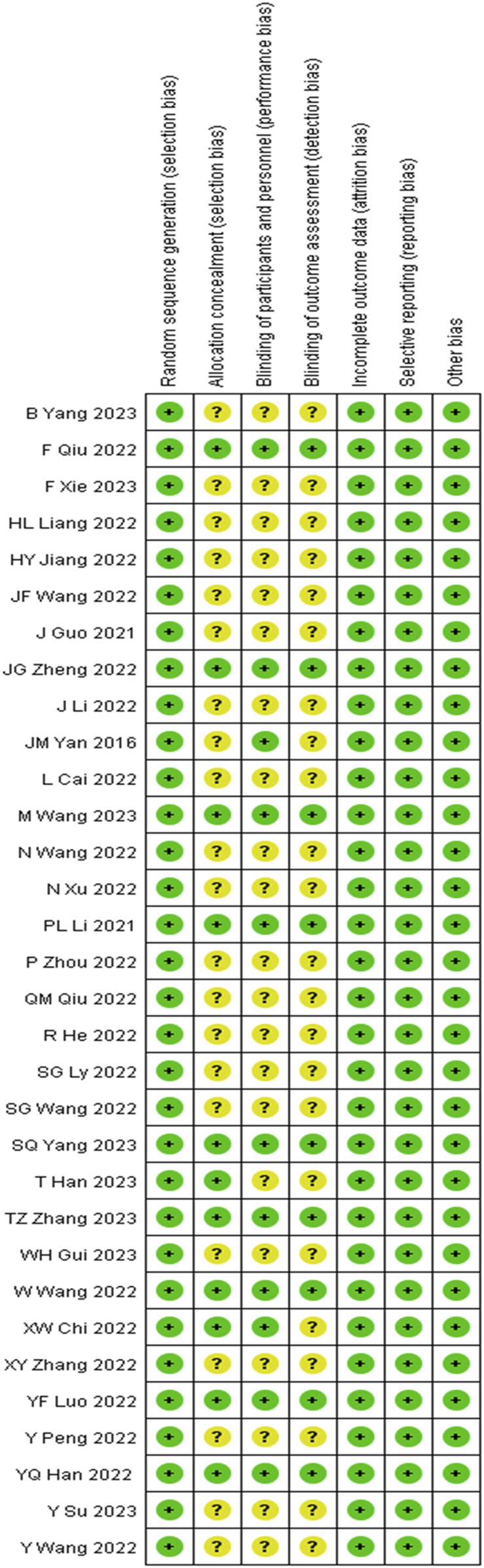
Risk of bias of the included studies, based on the Cochrane Risk of Bias tool.

All 32 studies (Guo et al., 2021; Li, 2021; Lyu et al., 2021; Yan, 2021; Wang JF. et al., 2022; Wang N. et al., 2022; Cai et al., 2022; Chi et al., 2022; Wang Y. et al., 2022; Wang W. et al., 2022; Han et al., 2022; He et al., 2022; Jiang et al., 2022; Li et al., 2022; Liang, 2022; Luo et al., 2022; Peng et al., 2022; Qiu et al., 2022; Qiu and Wang, 2022; Wang, 2022; Xu and Li, 2022; Zhang et al., 2022; Zheng et al., 2022; Zhou et al., 2022; Yang B. et al., 2023; Zhang et al., 2023a; Yang SQ. et al., 2023; Gui et al., 2023; Han et al., 2023; Su et al., 2023; Wang et al., 2023; Xie et al., 2023) described the details of random sequence generation. Twelve studies (Li, 2021; Yan, 2021; Chi et al., 2022; Wang W. et al., 2022; Han et al., 2022; Luo et al., 2022; Qiu and Wang, 2022; Zheng et al., 2022; Zhang et al., 2023a; Yang SQ. et al., 2023; Han et al., 2023; Wang et al., 2023) described the blinding method of participants and people and were thus considered to have a low risk of bias for this domain, while the remaining 20 studies did not describe the blinding methods and were considered to have an unclear risk of bias. Ten studies (Li, 2021; Chi et al., 2022; Wang W. et al., 2022; Han et al., 2022; Qiu and Wang, 2022; Zheng et al., 2022; Zhang et al., 2023a; Yang SQ. et al., 2023; Han et al., 2023; Wang et al., 2023) described the methods of assigning concealment. Detailed information on the methodological quality of the included studies is shown in Figure 2.

### 3.4 Results of meta-analysis

#### 3.4.1 Postoperative VAS score

**FIGURE 3:**
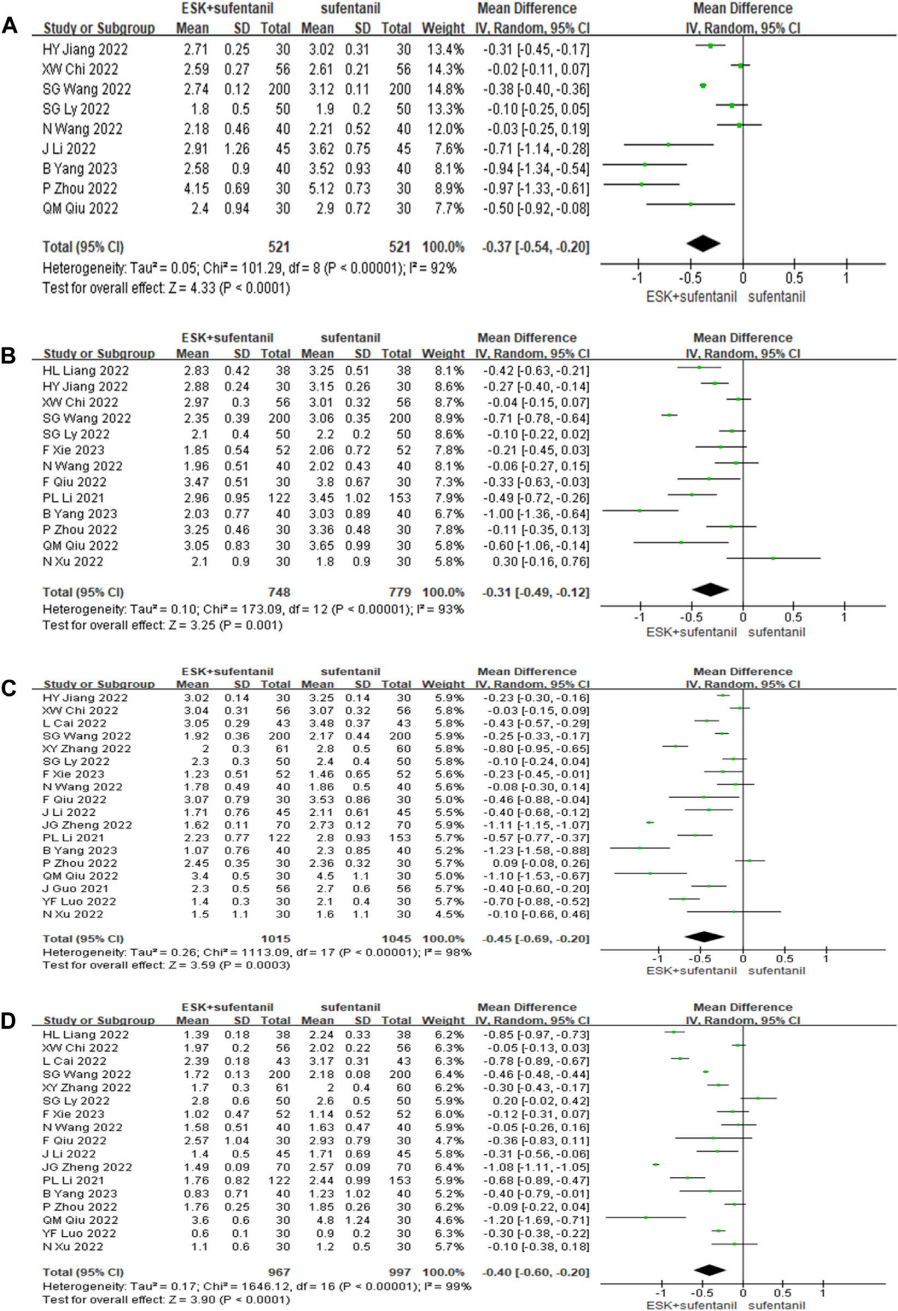
Forest plot of VAS scores at 6 h **(A)**, 12 h **(B)**, 24 h **(C)**, and 48 h **(D)** postoperatively. ESK = esketamine; VAS = visual analogue scale.

Eighteen studies reported VAS scores for esketamine combined with sufentanil and sufentanil alone at 6, 12, 24, and 48 h after surgery (Figure 3). The random effects model was used to analyse the pooled data. The outcomes indicated that the VAS scores for esketamine combined with sufentanil were significantly lower than those of sufentanil alone at 6, 12, 24, 48 h [MD_6_ = −0.37 points, *CI*: (−0.54, −0.20), *p* < 0.0001]; [MD_12 _= −0.31 points, *CI*: (−0.49, −0.12), *p *= 0.001]; [MD_24_ = − 0.45 points, *CI*: (−0.69, −0.20), *p* = 0.0003]; [MD_48_ = −0.40 points, *CI*: (−0.60, −0.20), *p* < 0.0001]. Therefore, the clinical effect of the combination of the two drugs for postoperative self-controlled intravenous analgesia at 6, 12, 24, and 48 h was significantly better than that of sufentanil alone. Sensitivity analysis showed that when study by SG Wang et al. was deleted, the heterogeneity decreased from 93% to 78% at 12 h, but the heterogeneity is still high in other time periods.

#### 3.4.2 Postoperative RSS score

**FIGURE 4:**
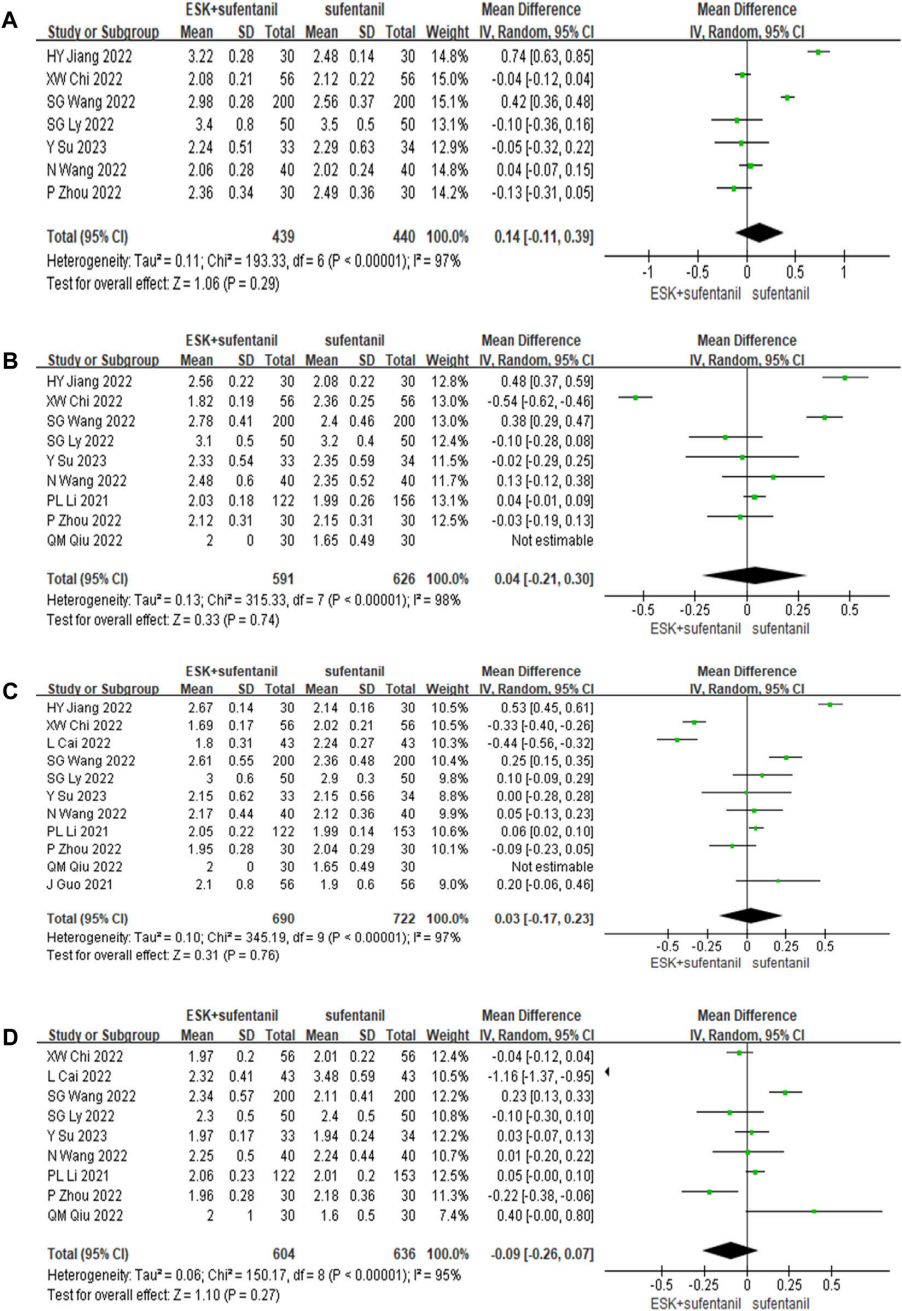
Forest plot of RSS scores at 6 h **(A)**, 12 h **(B)**, 24 h **(C)**, and 48 h **(D)** postoperatively. RSS = Ramsay Sedation Scale.

Eleven studies reported RSS scores for esketamine combined with sufentanil and sufentanil alone at 6, 12, 24, and 48 h after surgery. There was obvious heterogeneity among different studies (*p* < 0.01); this heterogeneity was potentially related to many factors, such as surgical methods, patients’ own differences, PCIA administration plans, and compatible doses. Therefore, the random effects model was used to analyse the pooled data. The results of the meta-analysis showed that the use of esketamine and sufentanil led to nonsignificantly higher RSS scores at 6, 12, 24, and 48 h after surgery than the use of sufentanil alone (*p* > 0.05). This finding indicates that esketamine combined with sufentanil had no significant effect on enhancing postoperative sedation, as shown in Figure 4. Sensitivity analysis was conducted and the results remained stable, while heterogeneity is still high.

#### 3.4.3 Postoperative adverse reaction rate

**FIGURE 5:**
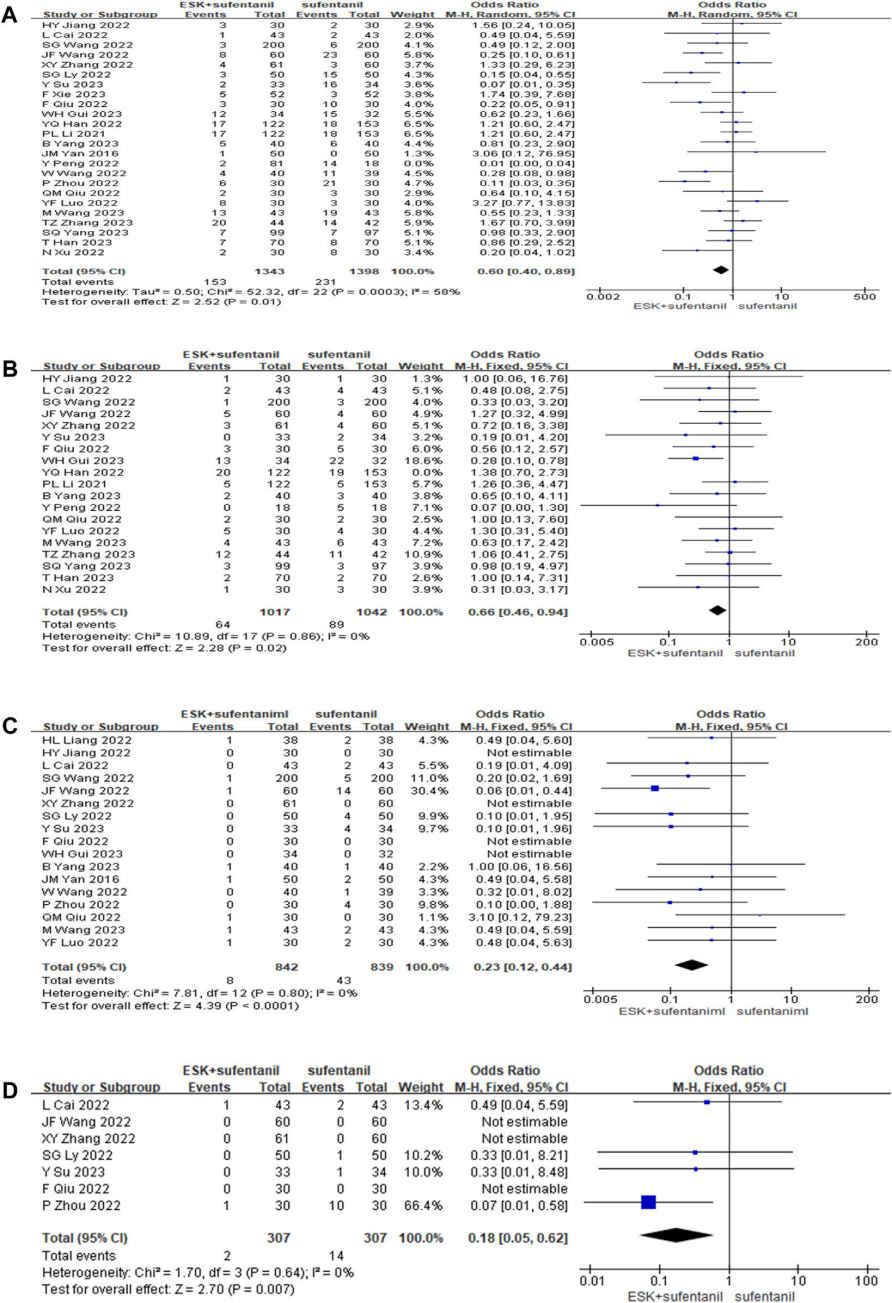
Forest plot of postoperative nausea, vomiting **(A)**; dizziness, headache **(B)**; itchy skin **(C)**; respiratory depression **(D)**.

Twenty-four studies reported the incidence of postoperative adverse events (Figure 5). The results showed that compared with patients treated with sufentanil alone, patients treated with esketamine-sufentanil combination therapy had lower incidence rates of PONV [OR = 0.60, *CI*: (0.40, 0.89), *p* = 0.01], dizziness-headache [OR = 0.66, *CI*: (0.46, 0.94), *p* = 0.02], pruritus [OR = 0.23, *CI*: (0.12, 0.44), *p* < 0.0001], and respiratory depression [OR = 0.18, *CI*: (0.05, 0.62), *p* = 0.007]. All outcomes showed significant differences between treatment groups. These findings indicate that the combination of esketamine and sufentanil significantly reduces the incidence of postoperative adverse reactions.

#### 3.4.4 Postoperative postpartum depression score

**FIGURE 6:**
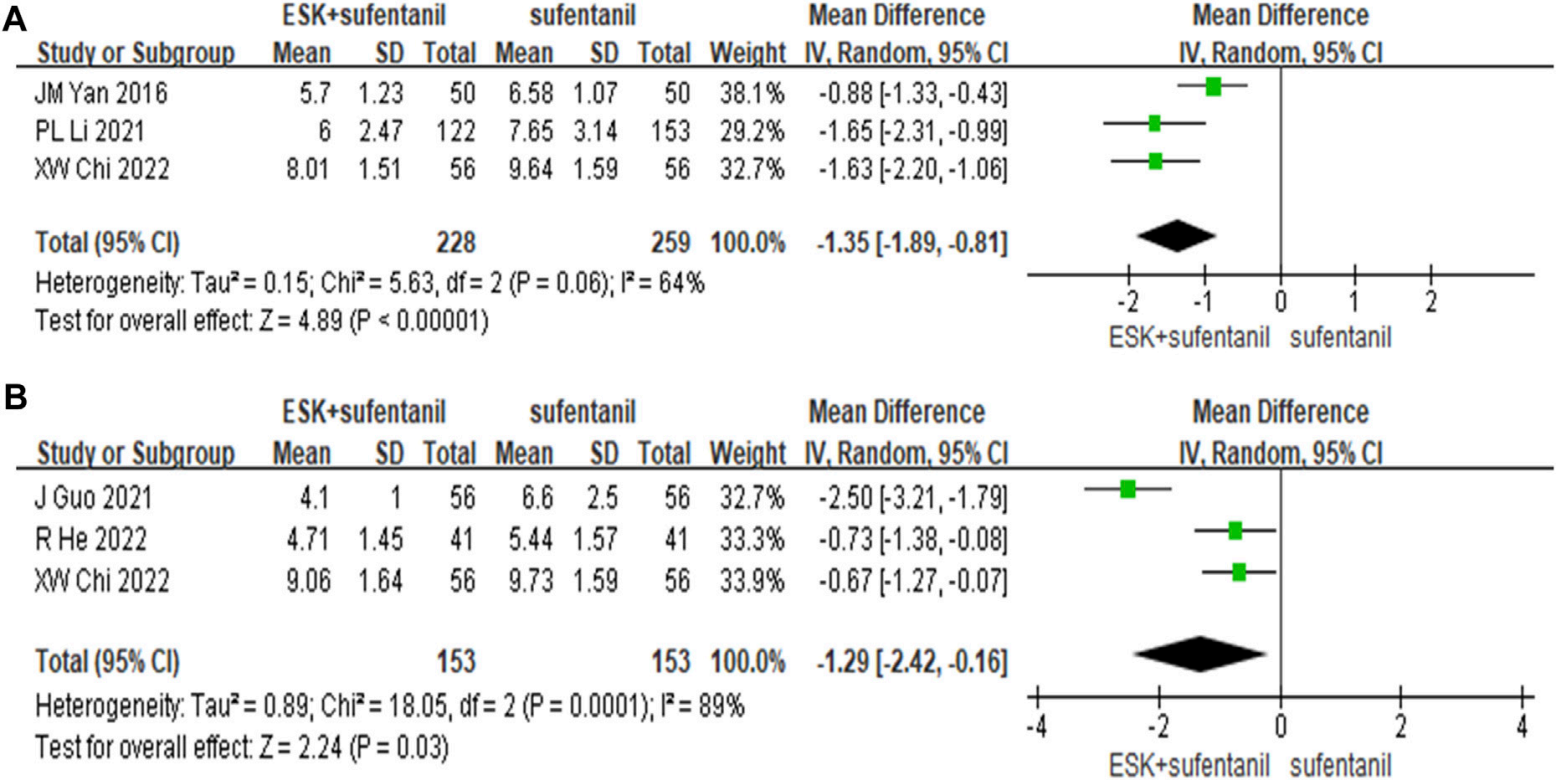
Forest plot of EPDS scores at 3 days **(A)** and 7 days **(B)** postoperatively. EPDS = Edinburgh postnatal depression scale.

The study analysis found that esketamine was widely used for caesarean section, so we performed subgroup analysis and collected the Edinburgh postpartum depression scores at 3 days and 7 days postpartum. We found that the differences between groups were statistically significant (Figure 6). The reduction in postpartum depression scores were greater in the esketamine-sufentanil group than in the sufentanil alone group at 3 days [MD = −1.35, *CI*: (−1.89, −0.81), *p* < 0.00001] and 7 days [MD = −1.29, *CI*: (−2.42, −0.16), *p* = 0.03]. These results show that esketamine-sufentanil can effectively reduce the incidence of postpartum depression.

#### 3.4.5 Publication bias

**FIGURE 7:**
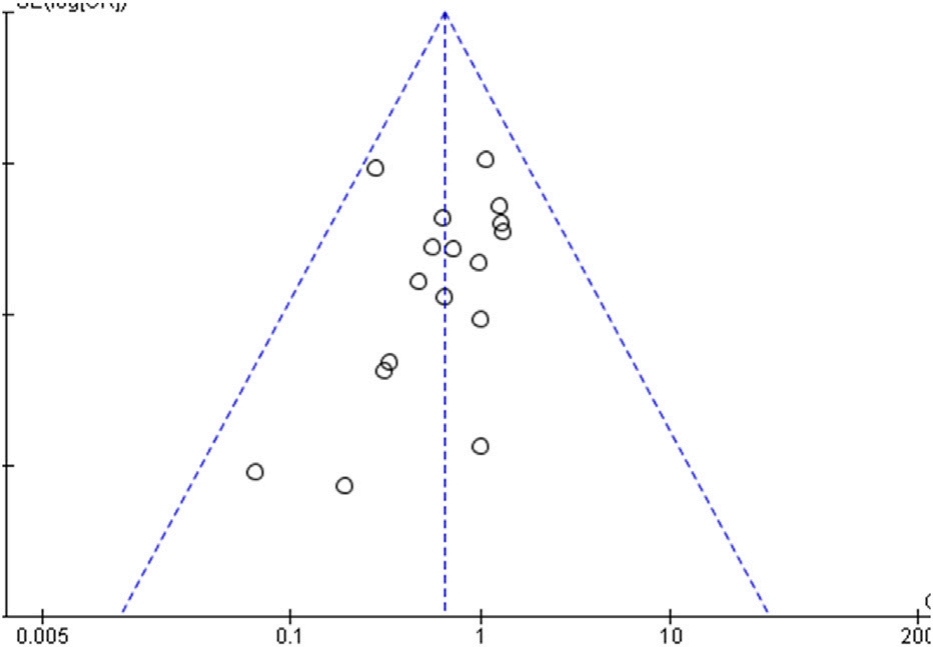
Funnel plot for publication bias assessment.

For the 32 included studies, we analysed the publication bias for the outcome of dizziness and headache by constructing a funnel plot. The results are shown in Figure 7, with the OR value as the centre, indicating that all sample points are scattered. This suggests that there was some publication bias, indicating that the literature may have a higher degree of clinical heterogeneity and publication bias.

## 4 Discussion

Postoperative pain is one of the most common complications in patients who undergo surgery, and effective postoperative analgesia is a requirement for patients to recover quickly after surgery. At present, PCIA is widely used clinically for postoperative analgesia, and the use of two or more kinds of analgesic drugs for PCIA can achieve good analgesic effects while reducing the drug dosage (Albrecht et al., 2016). Sufentanil, which is a derivative of fentanyl, is the most common postoperative analgesic drug in clinical practice at present. However, an increase in the dosage is associated with increases in nausea, vomiting and other adverse reactions (Choi et al., 2014). Esketamine, which is the S (+) isomer of ketamine, has a bioavailability of up to 100% when injected intravenously. Esketamine has 3–4 times the affinity of ketamine for NMDA receptors (Jelen et al., 2021) and 2–3 times the affinity of ketamine for opioid receptors (Zanos et al., 2018b). Although the incidence of dissociation symptoms and other psychotic adverse reactions caused by esketamine is higher than that of ketamine at the same dose and is dose dependent, the side effects caused by ketamine are somewhat related to the dose of ketamine. Furthermore, the dosage of esketamine is half of that of ketamine with the same analgesic effect. Therefore, the use of esketamine during anaesthesia produces a less irritating response to the patient’s heart, and the analgesic effect is better. Additionally, esketamine is helpful for alleviating the patient’s bad mood and can meet the analgesic requirements at a lower dose.

Chen et al. (2017) found that compared with the sufentanil group, the use of ketamine-sufentanil for PCIA not only reduced the analgesic effect but also reduced the incidence of PONV and other adverse reactions. Riddell et al. (2019) have also found that low-dose ketamine is an effective adjuvant to reduce pain and opioid demand during painful orthopaedic surgery, especially in the first 24 h after surgery. However, although the structure of esketamine is similar to that of ketamine, their pharmacokinetics and pharmacodynamics are different. Further analyses are needed to determine whether the combination of esketamine and sufentanil will have the same effect.

In this study, we analysed the role of esketamine as a sufentanil adjuvant in the treatment of PCIA and found that esketamine has a significant role in postoperative analgesia. In addition, the incidence of side effects related to sufentanil (such as nausea-vomiting, dizziness-headache, skin pruritus and respiratory depression) were reduced in the esketamine-sufentanil group. The combination of esketamine and sufentanil was also effective in reducing the incidence of postpartum depression.

Compared with the sufentanil group, the rate of complications in the esketamine-sufentanil group were significantly lower, and the sedation scores of patients were lower. The potential reasons for these phenomena are as follows. 1) The combination of the two drugs can reduce the use of sufentanil compared with a single-drug treatment. 2) There is a certain connection between the NMDA receptor and the opioid receptor on which esketamine acts. Animal studies have shown that NMDA receptor antagonists can reduce the incidence of adverse reactions such as nausea-vomiting by inhibiting the release of opioids (Patierno et al., 2005).

Zhang et al. (2023b) found that the esketamine-sufentanil combination for PCIA was highly effective in reducing postoperative pain at 24 h and significantly reduced the incidence of PONV compared with sufentanil alone. The results of the current study are consistent, thus providing a rationale for the administration of esketamine after 24 h. Previous meta-analyses usually analysed opioids (such as sufentanil, fentanyl, or morphine). Esketamine, as a new analgesic drug, has been widely used in the clinic in recent years. There are many studies on the commercial efficacy of esketamine in the treatment of PCIA in combination with sufentanil, but there is a lack of evidence-based medicine on the effects and safety. In this study, we integrated studies on the use of esketamine, with 28 publications in the last 7 years.

Our research revealed an article by YQ Han (Han et al., 2022). We contacted the author but did not receive any response, so we only included data on postoperative adverse reactions. This meta-analysis has some limitations. 1) Although the number of studies is large, most of them are of low methodological quality, and blinding methods were not implemented, leading to a high risk of bias and potentially affecting the results. 2) All of the included studies investigated Chinese adult patients, and even though they were published in English, it is unclear at present that our study results can be generalized to other racial groups. 3) The type of surgery, perioperative anaesthesia regimen, and drug doses varied across studies; thus, there was a high degree of heterogeneity. 4) This research did not evaluate the impact of various doses, and more randomized controlled trials are required to determine the best doses of esketamine and sufentanil for the various procedures.

Finally, in the preparation process of analgesic solutions, when two or more drugs are mixed together, visible physical reactions such as precipitation, discolouration, turbidity and gas production may occur due to the different physical and chemical properties of drugs or invisible chemical reactions such as hydrolysis, redox and titre reduction. Few studies have examined the stability compatibility of analgesic drugs in analgesic pumps. Therefore, it is necessary to strengthen the investigation and evaluation of the stability compatibility of esketamine and sufentanil in analgesic pumps to ensure the safety of clinical medication and reduce the occurrence of drug injury events.

## 5 Conclusion

Compared with sufentanil alone, the combination of esketamine and sufentanil for intravenous PCIA was more effective in terms of relieving pain, reducing the incidence of adverse effects, and decreasing the rate of postpartum depression, but there was no significant difference in sedation.
